# Engineering internally his-tagged ESX secretion system fusion antigens for tuberculosis nanoparticle vaccines

**DOI:** 10.1016/j.jbc.2026.113337

**Published:** 2026-07-16

**Authors:** Wen-Ling Hsu, Yang Jiao, Wei-Chiao Huang, Kristina N. Tran, Brennen T. Troyer, Matthew Hvasta, Brian Kuhlman, Andres Obregon-Henao, Jonathan F. Lovell

**Affiliations:** 1 Department of Biomedical Engineering, University at Buffalo, State University of New York, Buffalo, New York, USA; 2 Department of Microbiology, Immunology and Pathology, Colorado State University, Fort Collins, Colorado, USA; 3 Department of Biochemistry and Biophysics, University of North Carolina School of Medicine, Chapel Hill, North Carolina, USA

**Keywords:** tuberculosis, vaccines, protein, liposome, fusion protein, linker

## Abstract

Improved tuberculosis (TB) vaccines are needed to combat the disease, a leading cause of infectious disease death worldwide. The culture filtrate TB antigens ESAT6 and CFP10 form dimeric complexes, serve as immune markers for TB exposure and are vaccine antigen candidates. Here, we report that expressing an ESAT6-CFP10 fusion protein with a conventional glycine-serine (GS) linker sequence significantly enhanced expression yield in *E*. *coli* compared to the single antigens. Substituting the GS linker with an internal His-tag generated the ESAT(Int7)CFP fusion, retained elevated expression levels while simultaneously enabled purification with immobilized metal chromatography. The internal His-tag also permitted rapid protein coupling with cobalt-porphyrin (CoPoP) liposomes. Immunization with these constructs elicited antigen-specific antibodies and T cell responses and reduced lung bacterial burden in mice following aerosol TB challenge. Another pair of TB culture filtrate antigens, EsxH and EsxG yielded no soluble protein as individual antigens. However, when linked with an internal His-tag, high soluble expression was observed for EsxH(Int7)EsxG, and this fusion antigen also conferred partial protective immunity in TB challenged mice after vaccination with CoPoP. These findings support the design and manufacturability of multivalent TB vaccine candidates through engineered fusion antigens and nanoparticle delivery platforms.

Tuberculosis (TB), caused by *Mycobacterium tuberculosis* (Mtb), remains a leading global health threat, despite extensive use of the bacille Calmette-Guérin (BCG) vaccine, a live attenuated *Mycobacterium bovis* strain introduced in 1921. In 2023, about 10.8 million individuals developed TB, including 400,000 cases of drug-resistant TB, with incidence rates rising alongside global population growth (1). The escalating burden of drug-resistant TB underscores the urgent need for more effective vaccines. Since 2021, several vaccine candidates have entered clinical evaluation, including seven in Phase I, six in Phase II, and four in Phase III, according to ClinicalTrials.gov. These candidates span a broad spectrum of platforms, including live-attenuated (*e*.*g*., MTBVAC), inactivated (*e*.*g*., RUTI), subunit (*e*.*g*., M72/AS01E, H107e/CAF10b, GamTBvac, ID93+GLA-SE, and AEC/BC02), viral vector (*e*.*g*., Ad5-105K, Ad5-205K, and TB/Flu-05E), mRNA (*e*.*g*., BNT164a1 and BNT164b1), and recombinant BCG vaccines (*e*.*g*., VPM1002). However, setbacks in previous trials such as MVA85A and AERAS-422 (2, 3, 4) highlight the persistent gap between antigen design and protective efficacy. Most vaccine strategies aim to augment BCG-induced immunity, yet no candidate has demonstrated sufficient efficacy to replace BCG.

A major hurdle in TB vaccine development is that administration of antigens alone is often insufficient to induce a strong adaptive immune response (5). Adjuvants are therefore essential to enhance antigen-specific T-cell responses (6, 7). Several TB vaccine candidates incorporate adjuvant-loaded liposomal delivery systems to improve immunogenicity. For instance, the Phase III candidate M72/AS01E uses AS01, a liposomal formulation comprising the TLR4 ligand, monophosphoryl lipid A (MPLA), and the saponin QS21 (8, 9). Liposomal delivery facilitates lymphatic drainage of antigens to lymph nodes, enhances antigen retention, reduces degradation, and improves cross-presentation to CD8^+^ T cells *via* MHC-I pathways (10).

Recent research has also focused on Mtb virulence factors and their roles in host-pathogen interactions. Among the best characterized are ESAT6 (EsxA; Rv3875) and CFP10 (EsxB; Rv3874), secreted *via* the ESAT-6 Secretion System 1 (ESX-1), a Type VII secretion system. These proteins form a tight 1:1 heterodimeric complex critical to Mtb virulence and immune modulation (11, 12, 13, 14, 15, 16, 17). ESAT6 suppresses Toll-like receptor 2 (TLR2) signaling (18, 19) and modulates host cytokine responses and immune cell recruitment (20, 21, 22), while CFP10 promotes IFN-γ production, neutrophil recruitment, and cytolytic T cell activation (23, 24). Both antigens are absent from BCG owing to deletions within the region of deletion-1 (RD1) locus, yet they are highly immunogenic and widely used in diagnostics (*e*.*g*. QFT-Plus, T-SPOT) (25). A parallel secretion system, ESX-3, secretes another immunogenic dimeric pair, EsxG (TB9.8; Rv0287) and EsxH (TB10.4; Rv0288), involved in metal ion acquisition and immunogenicity (26, 27, 28, 29, 30, 31, 32, 33).

ESAT6, CFP10, EsxH, and EsxG have been incorporated into a range of TB vaccine candidates across diverse platforms, including viral vectors, adjuvanted protein subunits, and fusion protein vaccines. TB/Flu-01L, a replication-deficient influenza virus A vector expressing ESAT6, completed Phase I evaluation (ClinicalTrial.gov: NCT03017378), while TB/Flu-04L, co-expressing ESAT6 and Ag85A, has also completed Phase I trials, though subsequent trials are on hold (34, 35, 36). TB/FLU-05E, a recombinant attenuated influenza vector co-expressing EsxH and HspX within a truncated NS1 backbone, showed protection in preclinical models and completed a Phase II trial (37, 38). Another viral vector-based candidate, MVATG18598, uses a Modified Vaccinia Ankara vector to express five fusion proteins, including Rv2626c-T2A-Ag85B, CFP10-ESAT6, EsxH-EsxG, RpfB-RpfD and Rv3407-E2A-Rv1813c. In a post-exposure mouse model, MVATG18598 conferred greater protection than chemotherapy alone when combined with standard antibiotics (39). Other protein-based vaccines have shown similar promise. Recombinant ESAT6 adjuvanted with MPLA and dimethyl dioctadecylammonium bromide, ESAT6 combined with c-di-AMP (ESAT6:c-di-AMP) delivered intranasally, palmitoylated ESAT6 lipopeptides, and ESAT6 combined with CFP10 and proteoliposomes each elicited protective immunity in preclinical models (40, 41, 42, 43).

Several fusion protein vaccines, including H1 (Ag85B-ESAT6), H56 (Ag85B-ESAT6-Rv2660c), H4 (Ag85B-EsxH), and H28 (Ag85B-EsxH-Rv2660c), demonstrated efficacy in prophylactic models, while only ESAT6-containing fusions conferred post-exposure protection (44). H1/IC31 (H1 adjuvanted with IC31) and H56/IC31 have completed multiple Phase I/II trials (45, 46, 47, 48). However, H4/IC31, comprising Ag85B-EsxH fusion, failed to outperform BCG revaccination in Phase II trials, despite inducing strong T cell responses (49, 50, 51, 52, 53, 54). Additional fusion protein candidates include the quadrivalent HEHR (HSP90-ESAT6-HspX-RipA) delivered with CIA09A liposomes, and AEC/BC02 vaccine, comprising Ag85B and ESAT6-CFP10 with BC02 adjuvant, which was protective in a therapeutic model of latent TB but not prophylactically (55, 56). GamTBvac, containing dextran-binding domain-tagged Ag85B and ESAT6-CFP10 adjuvanted with CpG, and H107e/CAF10b, a multiepitope construct containing eight antigens (PPE68-ESAT6-EspI(Δ75-294)-ESAT6-EspC-ESAT6-EspA-ESAT6-MPT64-MPT70-MPT83) adjuvanted with CAF01 liposomes, are in clinical and preclinical development, respectively (57, 58, 59, 60, 61).

To preserve native antigenic structures, flexible glycine-serine linkers are frequently employed in ESAT6-CFP10 fusions (58, 62, 63), though alternative rigid linkers derived from Mtb peptides have also been used, as in MVATG18598, which uses residues of Rv1827 as the linkers of CFP10-ESAT6 and EsxH-EsxG fusions, respectively (39).

We have previously established a cobalt porphyrin-phospholipid (CoPoP)-based liposomal vaccine platform with broad applicability across infectious diseases, including malaria (64, 65, 66, 67), Lyme disease (68), and SARS-CoV-2 (COVID-19) (69, 70). CoPoP liposomes stably coordinate histidine-tagged recombinant antigens in serum, promoting efficient antigen display and eliciting robust, balanced humoral responses. Notably, the CoPoP-based SARS-CoV-2 vaccine (EuCorVac-19) has successfully advanced through Phase 2 and 3 clinical trials (71, 72, 73, 74). In our prior TB studies, an Ag85B mutant formulated with CPQ liposomal system (CoPoP/PHAD-3D6A/QS-21) induced strong antigen-specific antibody and T cell responses and conferred protection to mice against Mtb challenge without observable adverse effects on body weight (75).

The CPQ platform integrates well-characterized immunostimulatory adjuvants with complementary mechanisms of action. PHAD-3D6A, a synthetic monophosphoryl lipid A (MPLA) analogue and Toll-like receptor 4 (TLR-4) agonist, activates antigen-presenting cells while maintaining a non-pyrogenic profile (76, 77). QS-21, a saponin-based adjuvant, further amplifies immune responses by enhancing cytokine production and antibody responses when incorporated into the liposomal membrane (78). Comparative evaluations of CoPoP/PHAD-3D6A (CP) against a range of licensed and experimental adjuvants, including Alum, Titer Max, AdjuPhos, QuilA, Sigma Adjuvant System, Addavax (MF59-like), AS01, and complete/incomplete Freund’s adjuvant (CFA/IFA), demonstrated superior antigen-specific antibody induction with minimal local reactogenicity, highlighting the importance of antigen particulate display in driving potent immune activation (64). Addition of QS-21 to generate CPQ further enhanced immunogenicity, leading to markedly elevated antigen-specific IgG responses and increased frequencies of multifunctional (IL2^+^TNFα^+^IFNγ^+^) CD4^+^ and CD8^+^ T cells compared with CP or Alum (67).

Building on this platform, we engineered soluble ESAT6-CFP10 and EsxH-EsxG fusion proteins retaining native heterodimeric conformations, using glycine-serine linkers, further optimized with internal polyhistidine tags (Int7) to enhance yield, facilitate purification, and enable rapid CoPoP liposomal loading. Immunization with ESAT(Int7)CFP elicited high levels of antigen-specific antibodies and T cell responses, and significantly reduced lung bacterial burden in mice following aerosol TB challenge. Similarly, EsxH(Int7)EsxG vaccination reduced pulmonary Mtb burden in mice, supporting the use of native dimeric antigen pairs as next-generation vaccine components.

## Results

### Design and expression of the recombinant ESAT6, CFP10, and C-ESAT-CFP fusion proteins

**Figure 1:**
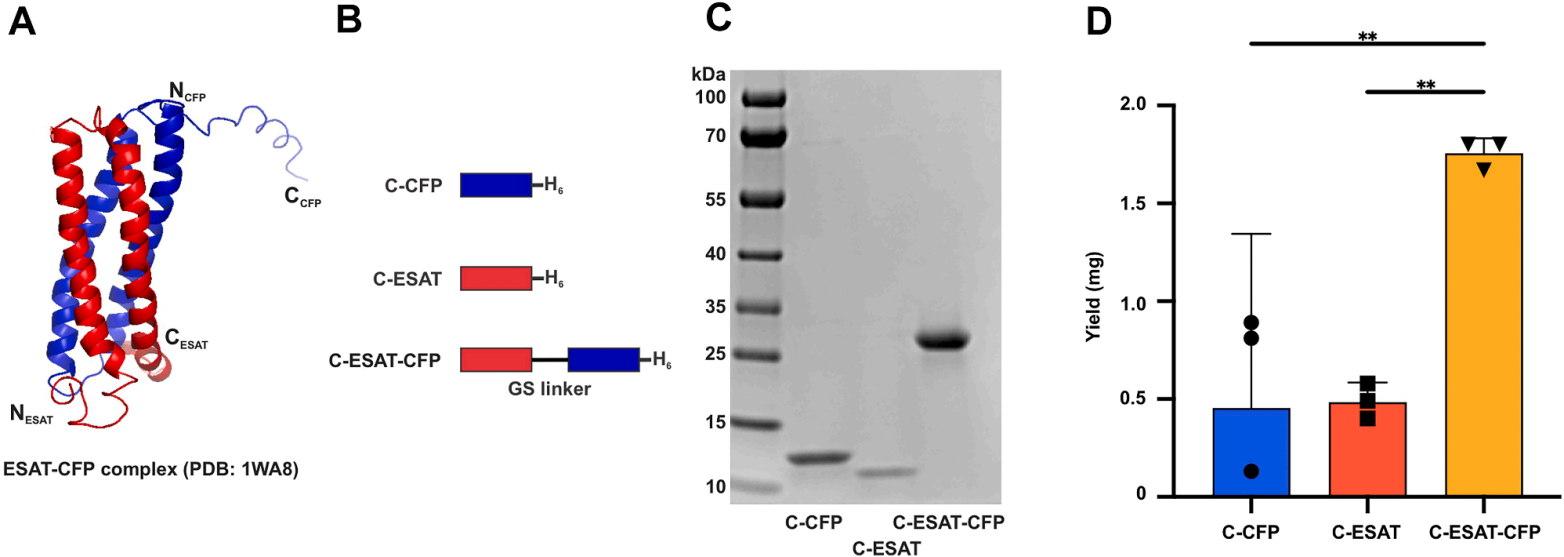
**Recombinant ESAT-CFP expresses better as a fusion compared to single antigen format.***A*, crystal structure of the ESAT-CFP protein complex (PDB code: 1WA8) with ESAT6 labeled in *red*, and CFP10 labeled in *blue*. (*B*) Design of the recombinant ESAT6 and CFP10 proteins. *C*, SDS-PAGE and (*D*) expression yields of the recombinant ESAT6 and CFP10 proteins in *E*. *coli* from 50 ml culture with standard procedure. Protein expression yields were determined by BCA assay following standard purification. The predicted size of C-CFP, C-ESAT, and C-ESAT-CFP are 12, 11, and 23 kDa, respectively. Graphs show mean ± std. dev. for n = 3 single colonies per group. Statistical significance was assessed using one-way analysis of variance (ANOVA) followed by Tukey’s test. Significance levels are indicated as ∗*p* < 0.05, ∗∗*p* < 0.01, ∗∗∗*p* < 0.005, and ∗∗∗∗*p* < 0.001.

To design the antigens, we examined the solution structure of the ESAT6-CFP10 complex (PDB ID: 1WA8), which revealed two helix-turn-helix hairpin structures formed by the individual proteins, with flexible and disordered termini (Fig. 1*A*). Guided by this structural insight, recombinant constructs of ESAT6 and CFP10 were designed with C-terminal His-tags (C-ESAT and C-CFP), and a fusion protein (C-ESAT-CFP) was engineered by linking ESAT6 and CFP10 *via* a 15-residue Gly-Ser repeat linker (GGGGSGGGGSGGGGS) (Fig. 1*B*). Expression in *E*. *coli* followed by Ni-NTA purification revealed that the soluble C-ESAT-CFP fusion protein (∼25 kDa) exhibited a higher expression level and greater purity (fewer background bands) than either C-CFP (∼12 kDa) or C-ESAT (∼11 kDa), as shown in the SDS-PAGE gels (Fig. 1*C*). Protein yields, determined by BCA assay, were 0.45 mg for C-CFP, 0.48 mg for C-ESAT, and 1.75 mg for C-ESAT-CFP per 50 ml culture (Fig. 1*D*). These results suggest that fusion of ESAT6 and CFP10 significantly enhanced soluble expression and yield, with more than a 3.5-fold increase compared to the individual proteins.

### Optimization of fusion protein expression using internal his-tag linker

**Figure 2:**
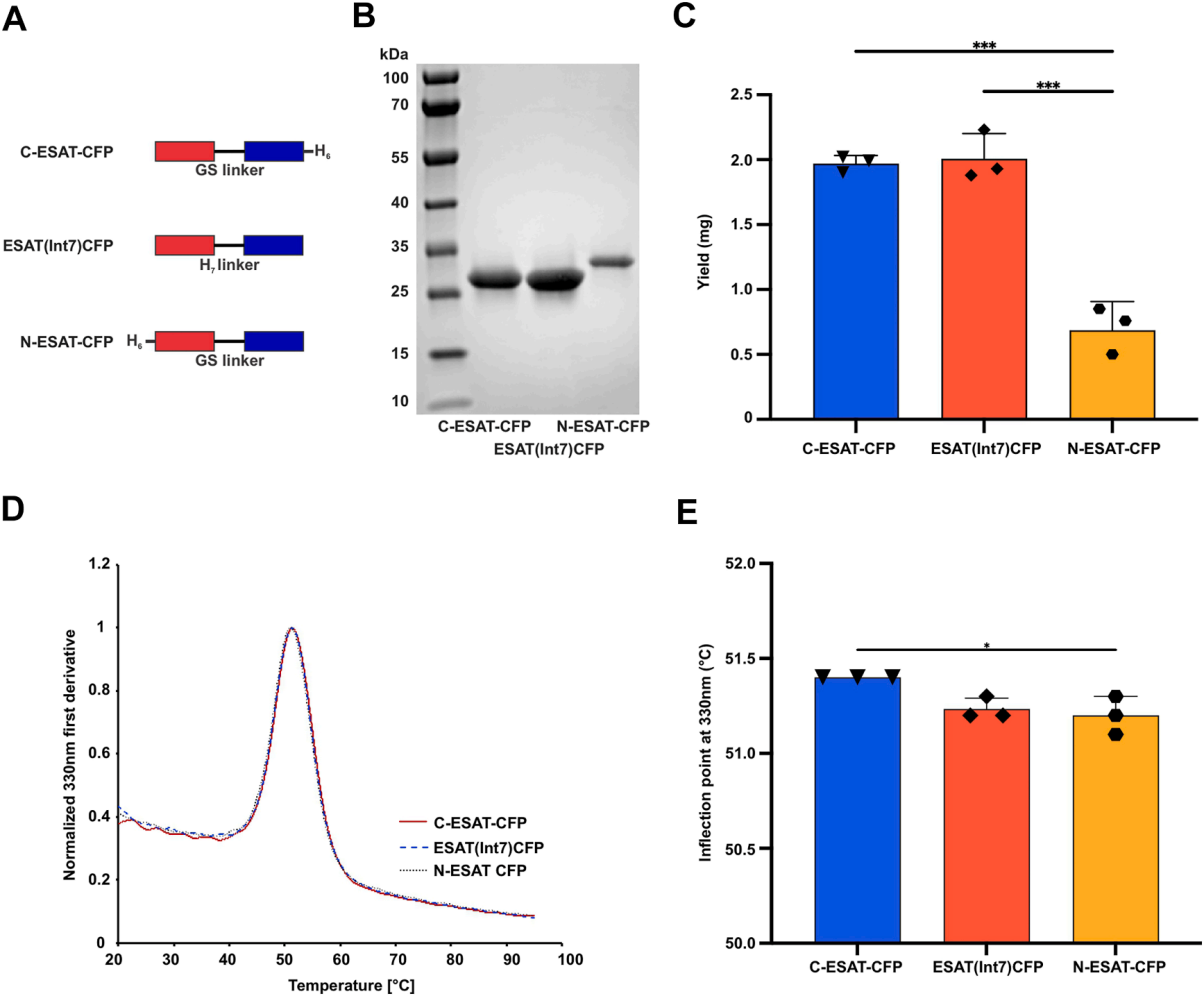
**Use of an internal his-tag linker for ESAT-CFP allows for high protein expression and downstream purification.***A*, Design of recombinant ESAT-CFP fusion proteins. *B*, SDS-PAGE and (*C*) expression yields of recombinant ESAT-CFP fusion proteins from 50 ml culture. Protein expression yields were determined by BCA assay following standard purification. The predicted size of C-ESAT-CFP, ESAT(Int7)CFP, and N-ESAT-CFP are 23, 23, and 23 kDa, respectively. *D*, thermal stability analysis of the ESAT-CFP fusion proteins. The melting curves were plotted by the normalized first derivative at 330 nm from the average of triplicates. *E*, the melting temperatures of the ESAT-CFP fusion proteins. Graphs show mean ± std. dev. for n = 3 measurements. Statistical significance was assessed using one-way analysis of variance (ANOVA) followed by Tukey’s test. Significance levels are indicated as ∗*p* < 0.05, ∗∗*p* < 0.01, ∗∗∗*p* < 0.005, and ∗∗∗∗*p* < 0.001.

To further enhance expression, we introduced an internal hepta-histidine (Int7) linker between ESAT6 and CFP10, generating the ESAT(Int7)CFP construct. An alternative construct, N-ESAT-CFP, was also created with an N-terminal His-tag and a GS linker (Fig. 2*A*). SDS-PAGE analysis confirmed high purity for all constructs. Among them, ESAT(Int7)CFP displayed the most intense band on the gel, while N-ESAT-CFP showed a slightly higher molecular weight shift compared to the others (Fig. 2*B*). Yields for ESAT(Int7)CFP (2.00 mg) and C-ESAT-CFP (1.97 mg) were notably higher than that of N-ESAT-CFP (0.69 mg) (Fig. 2*C*), suggesting that internal or C-terminal His-tag configurations are more favorable for ESAT-CFP fusion expression in *E*. *coli*. Consistent with these findings, expression analysis of individual ESAT6 and CFP10 proteins revealed that N-ESAT showed no detectable soluble expression (Fig. S1, *A* and *B*), whereas N-CFP exhibited lower expression levels than C-CFP (Fig. S1*C*). Therefore, these results suggest that N-terminal His-tag placement is unfavorable for soluble expression of ESAT-containing recombinant proteins in *E*. *coli* BL21 (DE3).

Thermal stability profiling revealed that all three constructs exhibited comparable melting temperatures (∼51.2-51.4 °C) (Fig. 2, *D* and *E*), indicating that they share similar structural stability. Altogether, these data suggest that the Int7 linker improved expression in *E*. *coli* without significantly compromising the stability of the fusion protein.

### Binding and structural integrity of ESAT-CFP on CPQ liposomes

**Figure 3:**
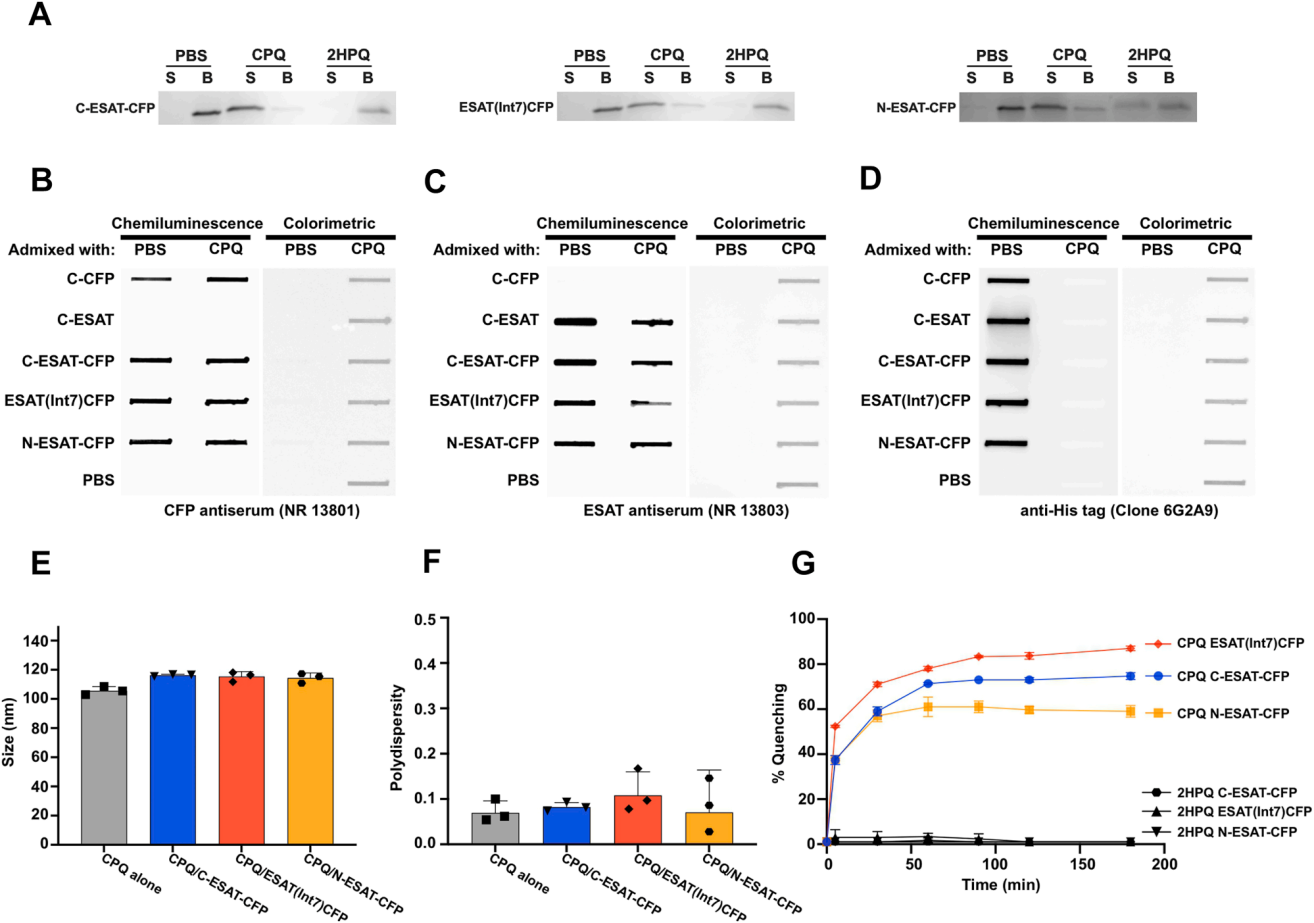
**Characterization of ESAT-CFP fusion protein binding to cobalt liposomes.***A*, Ni-NTA bead competition assay. Recombinant ESAT-CFP fusion proteins were incubated with liposomes for 3 h, followed by Ni-NTA bead isolation. Proteins captured by Ni-NTA beads (marked as “B”) *versus* liposome-bound proteins remaining in supernatant (marked as “S”). *B*, slot blot detection of recombinant ESAT-CFP fusion proteins binding to liposomes. CFP10 (*B*) and ESAT6 (*C*) proteins were identified with their respective antiserum. *D*, slot blot detection of the reactivity of the His-tag. His-tags were detected by monoclonal anti-His-tag antibodies. *E*, size measurement and (*F*) polydispersity liposomes following ESAT-CFP binding using dynamic light scattering analysis. *G*, kinetics of fluorescence quenching showing DY490-labeled ESAT-CFP protein binding to liposomes. Graphs show mean ± std. dev. for n = 3 measurements.

To assess the particulate vaccine potential, we evaluated the binding of His-tagged fusion proteins to CPQ liposomes. A Ni-NTA bead competition assay was used to analyze their ability to form particles with CPQ liposomes *via* cobalt-histidine interaction. Control liposomes, 2HPQ, which lack cobalt in the porphyrin ring, were also included. SDS-PAGE analysis of the assay showed that most ESAT-CFP fusion proteins were detected predominantly in the supernatant fractions of the CPQ groups, indicating strong liposome-binding affinity. In contrast, N-ESAT-CFP exhibited slight binding to 2HPQ liposomes (Fig. 3*A*).

Slot blot analysis confirmed that all CFP10-containing recombinant proteins, whether free or bound to CPQ, were recognized by the anti-CFP10 antiserum in the chemiluminescence mode. The presence of CPQ liposomes was verified by their characteristic green color in the colorimetric mode (Fig. 3*B*). Conversely, ESAT6-containing recombinant proteins were recognized by the anti-ESAT6 antiserum (Fig. 3*C*). Notably, His-tags on the proteins in PBS were detectable by the anti-His-tag antibody, whereas those bound to CPQ liposomes were not (Fig. 3*D*), suggesting that the His-tags were fully incorporated into the liposome membrane. These results confirm the conformational integrity of all CPQ-bound ESAT-CFP proteins, with preserved recognition by anti-ESAT6 and anti-CFP10 antisera, along with the loss of His-tag signal upon binding to CPQ liposome.

Dynamic light scattering analysis revealed a particle size shift from ∼106 nm (CPQ alone) to ∼114 to 116 nm post-protein binding, with low polydispersity (Fig. 3, *E* and *F*). A fluorescence quenching assay further demonstrated rapid particle formation, with ∼80% binding of labeled ESAT(Int7)CFP, ∼70% binding of C-ESAT-CFP, and ∼60% binding of N-ESAT-CFP within 90 min (Fig. 3*G*).

### CPQ/ESAT-CFP vaccines elicit humoral and cellular immunity in mice

**Figure 4:**
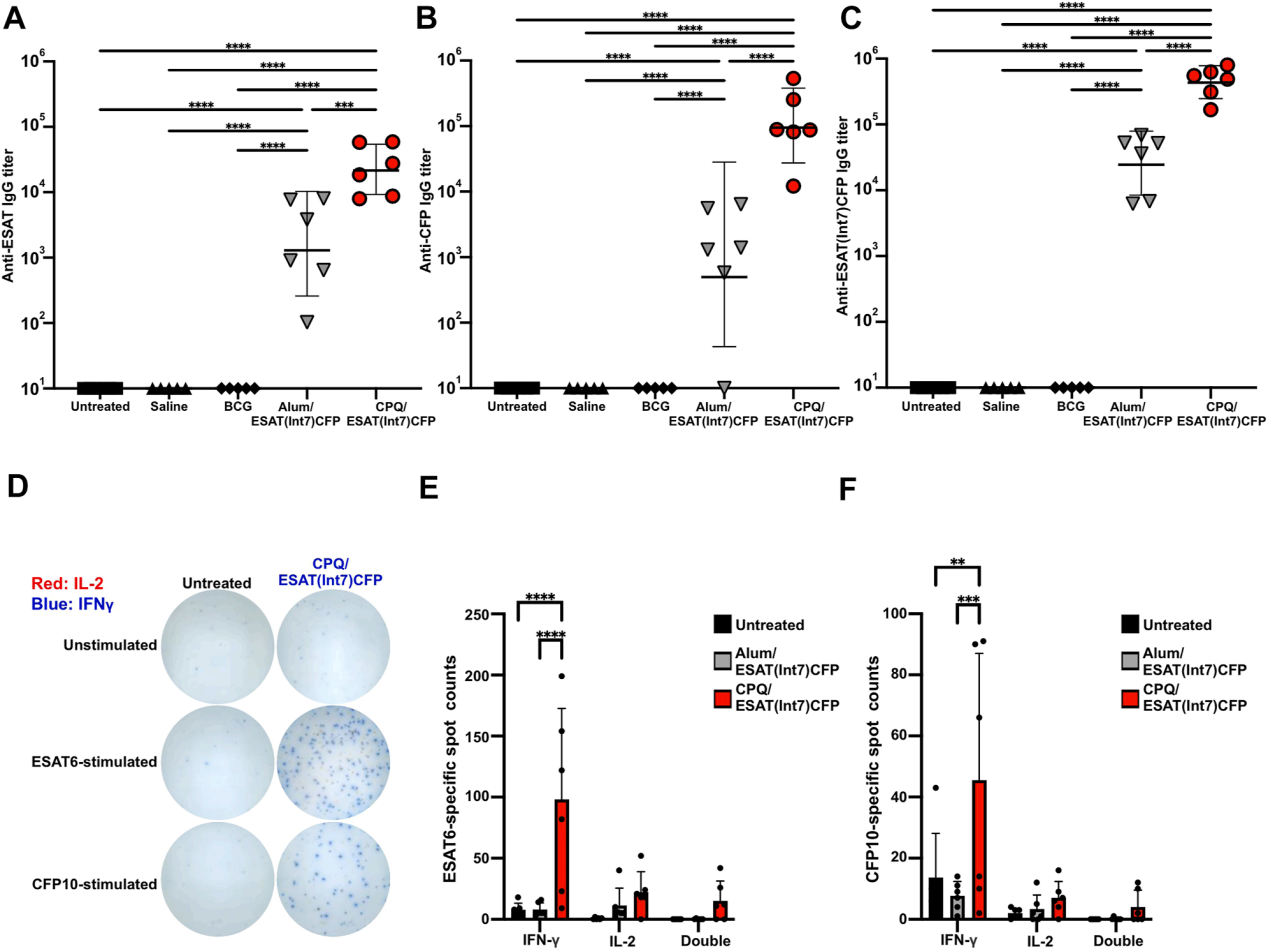
**CPQ/ESAT(Int7)CFP vaccines induce antigen-specific antibody and T cell responses in mice.***A–C*, CB6F1/J mice (n = 6 per group; n = 5 for saline and BCG) were immunized intramuscularly with ESAT(Int7)CFP vaccines (2 μg of antigen) on days 0 and 14. For BCG vaccination, mice were injected SubQ with 50 μl of 1 × 10^6^ CFU BCG Pasteur in the scruff of the neck and received a saline boost. Serum samples were collected on day 28 for assessment of anti-ESAT6, anti-CFP10, and anti-ESAT(Int7)CFP IgG titer by ELISA. Splenocytes were harvested 14 days post-second immunization and restimulated with the ESAT6 and CFP10 peptide pools. *D*, representative IFNγ/IL2 double-color ELISpot results. *E* and *F*, quantitative analysis of the single-positive (IFNγ or IL2) and double-positive (IFNγ^+^IL2^+^) spots. Data are presented as (*A–C*) geometric mean ± geometric SD and (*E* and *F*) mean ± SD (n = 6 per group). Statistical significance was assessed using one-way ANOVA followed by Tukey’s test. Significance levels are indicated as ∗*p* < 0.05, ∗∗*p* < 0.01, ∗∗∗*p* < 0.005, and ∗∗∗∗*p* < 0.001.

In our previous work, we demonstrated that a 2 μg antigen dose elicits robust immunogenicity in CoPoP-based vaccines targeting malaria and SARS-CoV-2 (67, 70, 79). In a pilot *M*. *tuberculosis* challenge study, we evaluated BCG administered at the standard dose as well as at 30- and 900-fold dilutions, and CPQ/ESAT(Int7)CFP and CPQ/EsxH(Int7)EsxG administered at antigen doses of 0.5 μg or 1.5 μg (Fig. S2). Immunization with 1.5 μg of antigen conferred significantly greater protection than the 0.5 μg dose for both fusion protein vaccines. Based on these findings, we selected a 2 μg antigen dose for subsequent vaccine formulations. The doses of CoPoP, PHAD-3D6A, and QS-21 in the CPQ formulation were 8, 3.2, and 3.2 μg, respectively. CB6F1/J mice were immunized intramuscularly with 2 μg of ESAT(Int7)CFP adjuvanted with either CPQ liposomes or Alum on day 0 and 14, and immunogenicity was evaluated on day 28. BCG-vaccinated mice received 1 × 10^6^ CFU BCG Pasteur subcutaneously and a saline boost. The CPQ/ESAT(Int7)CFP formulation induced significantly higher IgG titers against ESAT6, CFP10, and ESAT(Int7)CFP (∼4.3 × 10^4^, ∼5 × 10^5^, and ∼5.6 × 10^5^, respectively) than the Alum-adjuvanted group (∼3.1 × 10^3^, ∼2.7 × 10^2^, and ∼4.4 × 10^4^, respectively) as well as BCG, saline, and untreated controls (<10) (Fig. 4, *A* and *B*, *C*). To further characterize antigen-specific T-cell responses, IFNγ/IL2 double-color ELISpot assay was performed using ESAT-6 or CFP-10 peptide pools for restimulation. IFNγ/IL2 double-color ELISpot assays showed that immunization with CPQ/ESAT(Int7)CFP elicited significant increase in IFN-γ producing splenocytes and a modest increase in IL-2 producing splenocytes compared with both the Alum and control groups (Fig. 4, *D* and *E*, *F*). To further delineate the contribution of CPQ-mediated antigen display, an independent immunization study was performed comparing Alum/ESAT(Int7)CFP, 2HPQ/ESAT(Int7)CFP, and CPQ/ESAT(Int7)CFP formulations. IFNγ single-color ELISpot analysis using combined ESAT-6 and CFP-10 peptide pools demonstrated that CPQ/ESAT(Int7)CFP generated the highest levels of IFN-γ-positive splenocytes among all groups tested (Fig. S3*A*).

Collectively, these findings demonstrate that CPQ-formulated ESAT(Int7)CFP induces potent antigen-specific humoral and T cell immune responses, supporting the capacity of CPQ-mediated particulate antigen display to enhance immunogenicity.

### CPQ/ESAT-CFP vaccines induced short term protection against *M*. *tuberculosis* challenge in mice

**Figure 5:**
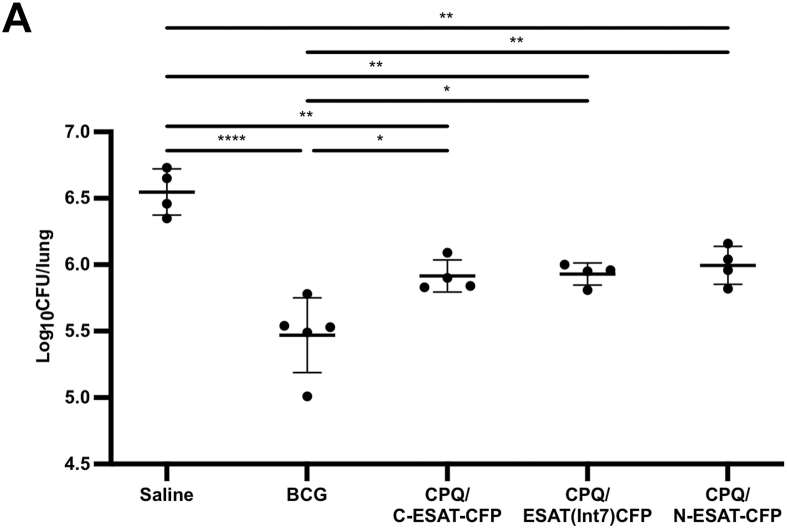
**Efficacy of immunization using CPQ/ESAT-CFP vaccine against *M*. *tuberculosis* challenge in mice.***A*, lung colony-forming units (CFUs). Graphs show mean ± std. dev. for n = 4 mice per group (n = 5 for BCG group). Data were analyzed using one-way ANOVA followed by Tukey’s test. ∗*p* < 0.05, ∗∗*p* < 0.01, ∗∗∗*p* < 0.005, and ∗∗∗∗*p* < 0.001.

To assess protective efficacy, immunized mice were challenged with *M*. *tuberculosis*, and lung bacterial burden was quantified 30 days post-challenge. The CPQ/ESAT(Int7)CFP, CPQ/C-ESAT-CFP, and CPQ/N-ESAT-CFP vaccines significantly reduced bacterial loads (5.93, 5.92, and 6.0 Log_10_ CFU/lung, respectively) compared to unimmunized controls (saline) (6.55 Log_10_ CFU/lung) (Fig. 5*A*). Although BCG-immunized mice showed the greatest protection (5.47 Log_10_ CFU/lung), mice immunized with CPQ/ESAT(Int7)CFP also exhibited reduced CFU counts and a smaller standard deviation compared with the BCG group.

### Expression of EsxH-EsxG fusion proteins

**Figure 6:**
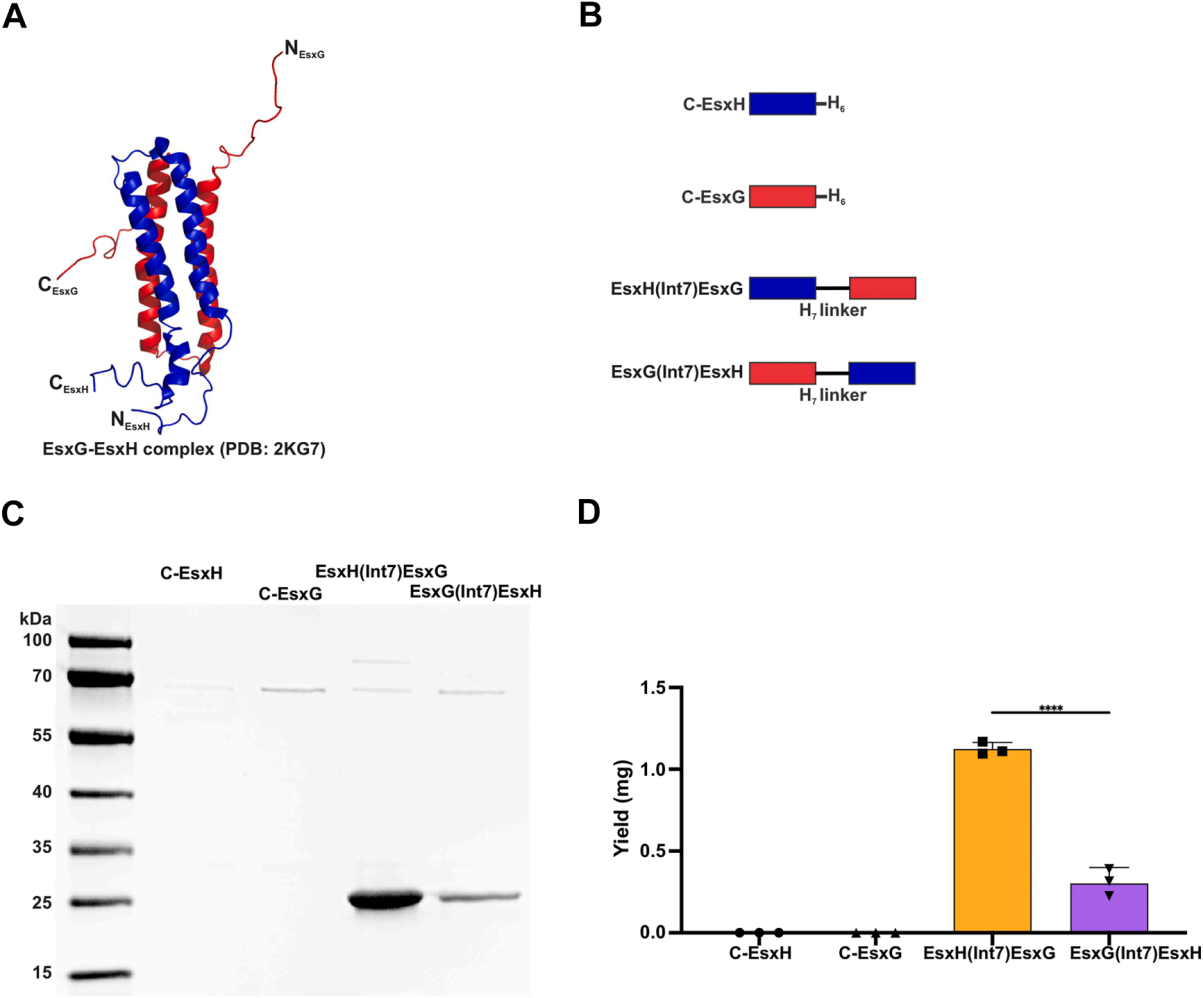
**Characterization of recombinant EsxH-EsxG fusion proteins.***A*, solution structure of the EsxG-EsxH protein complex (PDB ID: 2KG7). *B*, design of recombinant EsxG, EsxH, and EsxH-EsxG fusion proteins. *C* and *D*, SDS-PAGE and expression yields of the recombinant EsxH, EsxG, and EsxH-EsxG fusion proteins from 50 mL of culture. Protein expression yields were determined by BCA assay following standard purification. The predicted size of C-EsxH, C-EsxG, EsxH(Int7)EsxG, and EsxG(Int7)EsxH are 11, 11, 22, and 22 kDa, respectively. Graphs show mean ± std. dev. for n = 3 single colonies per group. Data were analyzed using one-way ANOVA followed by Tukey’s test. ∗*p* < 0.05, ∗∗*p* < 0.01, ∗∗∗*p* < 0.005, and ∗∗∗∗*p* < 0.001.

Building on the ESAT-CFP platform, we aimed to generate constructs for another antigen pair, EsxH and EsxG, based on their dimeric structure (PDB ID: 2KG7) (Fig. 6*A*). Their solution structure reveals that EsxH-EsxG complex resembles the ESAT-CFP complex, as both share a characteristic helical structure and form a barrel-like arrangement. We generated the fusion constructs EsxH(Int7)EsxG and EsxG(Int7)EsxH, along with the individual EsxH and EsxG proteins (Fig. 6*B*). Expression of the individual C-terminal His-tagged antigens (C-EsxH and C-EsxG) results in negligible amounts of soluble protein, with only faint background bands visible on the SDS-PAGE gel. In contrast, the fusion constructs EsxH(Int7)EsxG and EsxG(Int7)EsxH were expressed at high levels and in soluble form, yielding of 1.12 mg and 0.3 mg of protein, respectively (Fig. 6, *C* and *D*).

### Immunogenicity and protective efficacy of CPQ/EsxH-EsxG vaccines

**Figure 7:**
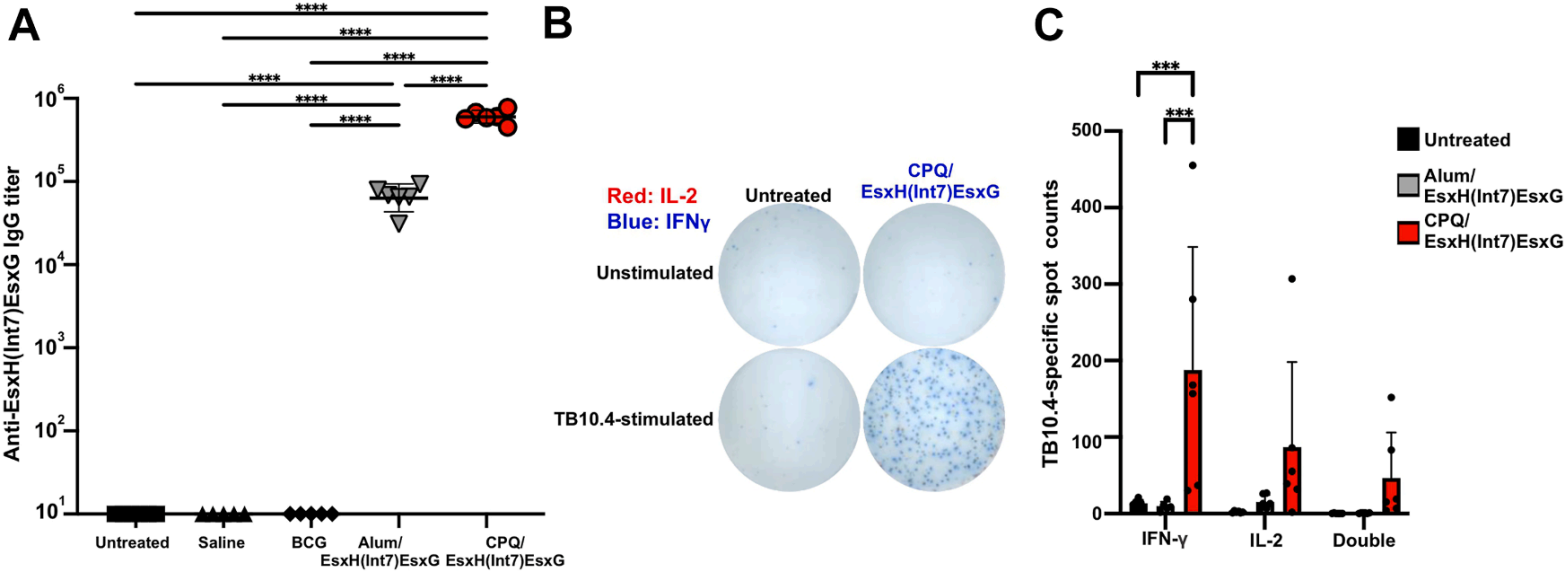
**CPQ-EsxH(Int7)EsxG vaccines induce antigen-specific antibody and T cell responses in mice.***A*, CB6F1/J mice were immunized intramuscularly with EsxH(Int7)EsxG vaccines (2 μg of antigen) on days 0 and 14. For BCG vaccination, mice were injected SubQ with 50 μl of 1 × 10^6^ CFU BCG Pasteur in the scruff of the neck and received a saline boost. Serum samples were collected on day 28 for assessment of anti-EsxH(Int7)EsxG IgG titer by ELISA (n = 6 per group; n = 5 for saline and BCG). Splenocytes from CB6F1/J mice (n = 6) vaccinated as mentioned above and restimulated with the TB10.4 peptide pool. *B*, representative IFNγ/IL2 double-color ELISpot results. *C*, quantitative analysis of the single-positive (IFNγ or IL2) and double-positive (IFNγ^+^IL2^+^) spots. Data are presented as (*A*) geometric mean ± geometric SD or (*C*) mean ± SD (n = 6 per group). Statistical significance was assessed using one-way ANOVA followed by Tukey’s test. Significance levels are indicated as ∗*p* < 0.05, ∗∗*p* < 0.01, ∗∗∗*p* < 0.005, and ∗∗∗∗*p* < 0.001.

CB6F1/J mice were immunized with 2 μg of EsxH(Int7)EsxG formulated with either CPQ liposomes or Alum using the same immunization regimen described above. Immunization with CPQ/EsxH(Int7)EsxG elicited significantly stronger antigen-specific IgG responses against EsxH(Int7)EsxG (∼5.8 × 10^6^) compared with the Alum-adjuvanted group (∼4.8 × 10^5^). In contrast, minimal antibody responses were observed in BCG-vaccinated, saline-treated, and untreated control mice (<10) (Fig. 7*A*).

To evaluate antigen-specific T-cell responses, splenocytes were restimulated with EsxH peptide pools and analyzed by IFNγ/IL2 double-color ELISpot assay. CPQ/EsxH(Int7)EsxG immunization elicited pronounced increase in IFN-γ-secreting splenocytes together with a modest elevation in IL-2-producing cells relative to Alum and control groups (Fig. 7, *B* and *C*), consistent with enhanced antigen-specific T cell activation. To further define the contribution of CPQ-mediated antigen presentation, an independent immunization compared Alum/EsxH(Int7)EsxG, 2HPQ/EsxH(Int7)EsxG, and CPQ/EsxH(Int7)EsxG formulations. IFNγ single-color ELISpot analysis with EsxH peptide pool restimulation demonstrated that CPQ/EsxH(Int7)EsxG generated the highest levels of IFN-γ-positive splenocytes among all groups examined (Fig. S3*B*).

**Figure 8:**
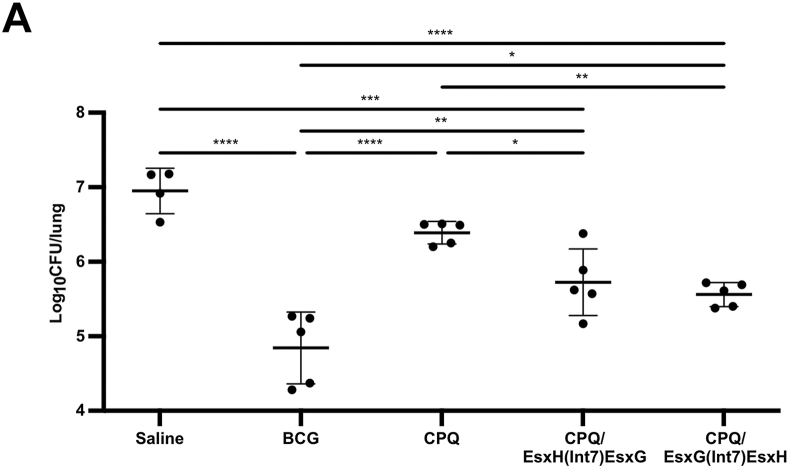
**CPQ/EsxH(Int7)EsxG and CPQ/EsxG(Int7)EsxH vaccines induce protection against *M*. *tuberculosis* challenge in mice.***A*, Lung colony-forming units (CFUs). Graphs show mean ± SD. for n = 5 mice per group (n = 4 for saline group). Data were analyzed using one-way ANOVA followed by Tukey’s test. ∗*p* < 0.05, ∗∗*p* < 0.01, ∗∗∗*p* < 0.005, and ∗∗∗∗*p* < 0.001.

Protective efficacy was subsequently assessed following aerosol *M*. *tuberculosis*. Immunization with either EsxH(Int7)EsxG or EsxG(Int7)EsxH formulated with CPQ significantly reduced lung bacterial burden (5.73 and 5.56 Log_10_ CFU/lung, respectively) compared to untreated controls (6.95 Log_10_ CFU/lung) and unadjuvanted CPQ liposomes (6.39 Log_10_ CFU/lung) (Fig. 8*A*). Among the fusion constructs tested, EsxG(Int7)EsxH exhibited the most comparable protective efficacy to BCG (4.84 Log_10_ CFU/lung), while also displaying lower inter-animal variability than the BCG group.

Together, these findings demonstrate that CPQ-formulated EsxH-EsxG fusion vaccines induce potent antigen-specific humoral and cellular immune responses and confer significant protection against pulmonary *M*. *tuberculosis* infection.

## Discussion

Liposomes have emerged as versatile delivery vehicles in modern vaccinology, with widespread application in both nucleic acid and protein-based vaccines. Beyond their success in mRNA vaccine platforms, such as those developed against SARS-CoV-2, liposomal nanoparticles are increasingly employed in subunit vaccines to enhance antigen stability, immunogenicity, and cost-efficiency. In our previous work, our group demonstrated that CPQ liposomes effectively stabilize recombinant antigens and elicit potent, balanced neutralizing antibody responses in malaria (64, 65, 66, 67), Lyme disease (68), and SARS-CoV-2 (COVID-19) (69, 70, 74). In addition to enhancing antigen stability and immunogenicity, the CPQ platform reduces vaccine formulation time and cost, as only microgram-scale quantities of antigen are required for effective liposomal display.

A feature of the present study is the use of an internal polyhistidine tag as part of the fusion architecture linking antigen pairs that naturally form heterodimeric complexes in Mtb. To our knowledge, this represents the first application of an internal His-tag strategy for linking TB fusion antigens. In contrast to conventional N- or C-terminal affinity tags, the internal His-tag simultaneously enabled recombinant purification and CoPoP-mediated liposomal binding without the need for tag removal, as the tags become sequestered within the liposomal bilayer upon particle formation. Additionally, the glycine-serine linker could be completely replaced by the functional His-tag. Mechanistically, this design allows liposome anchoring through the centrally positioned His-tag while leaving the flanking antigen domain exposed, potentially enhancing their accessibility for immune recognition. Furthermore, by linking antigen pairs that naturally associate in Mtb, this fusion architecture may better preserve native structural features and conformational epitopes than individually expressed antigens, although the extent to which such features are maintained remains to be determined. Notably, incorporation of the internal His-tag was also compatible with soluble expression of both the ESAT6-CFP10 and EsxH-EsxG fusion proteins.

A related strategy has previously been described for the αβ-thermosome complex from *Thermoplasma acidophilum*, in which a hexahistidine-tag was inserted into a loop region of the α subunit because both the N- and the C-terminal are positioned internally within the assembled protein complex, making terminal His-tag placement impractical (80). We speculate that the improved soluble expression observed in our constructs may reflect preservation of structurally important terminal regions required for heterodimer assembly and protein folding. In this context, conventional terminal His-tag may disrupt native intermolecular interactions or reduce protein solubility, whereas internal His-tag placement may maintain structural integrity while still permitting efficient purification and CoPoP-mediated particle formation. Although the precise molecular mechanisms remain to be defined, these findings suggest that internal His-tag engineering may provide a useful strategy for improving the manufacturability of structurally sensitive multimeric vaccine antigens.

A potential consideration of the present fusion strategy is the possibility of immune responses directed against the internal polyhistidine tag. Although affinity tags can theoretically introduce additional immunogenic epitopes, slot blot analysis performed after antigen loading onto CPQ liposomes showed no detectable reactivity with monoclonal anti-His-tag antibodies (Fig. 3*D*), suggesting that the internal His-tag becomes effectively incorporated within the CoPoP liposomal membrane and is not readily exposed for immune recognition. These findings imply that CoPoP-mediated particleization may partially shield the His-tag in the final vaccine formulation. Nevertheless, because direct assessment of anti-His-tag antibody responses was not performed in the present study, future investigations examine potential tag-specific immunity will be important to further establish the immunological properties of this platform. Due to the buried nature of the His-tag, anti-His-tag antibodies were found diminished with CoPoP presentation in preclinical studies (81) and in human testing (71).

In the early stages of this study, we observed that the individual ESAT6 and CFP10 antigens were difficult to express as soluble recombinant proteins at sufficient yield, limiting their practical utility for scalable vaccine manufacturing. This challenge motivated the development of fusion protein constructs, which substantially improved protein yield, purity, and ease of downstream purification. A similar limitation was observed for the individual EsxH and EsxG proteins, which showed poor soluble expression and were difficult to purify in bulk (Fig. 6*C*), increasing both technical complexity and production cost. These findings collectively support the use of fusion protein engineering as a practical strategy to improve recombinant TB antigens manufacturability. Accordingly, the present study focused primarily on the immunogenicity and protective efficacy of optimized fusion protein formulations.

Another limitation of this study is that we did not directly compare the fusion protein vaccines with vaccines formulated using the corresponding single antigens, such as ESAT, CFP, EsxH or EsxG alone. Such comparison would help further define whether fusion protein engineering provides immunological advantages beyond improved manufacturability. Considering the poor soluble yield of the single antigen constructs, inclusion body refolding approaches might need to be adopted for some of the antigens. Future studies should evaluate the relative immunogenicity, antigen presentation, and protective efficacy of single-antigen and fusion-protein formulations, as well as investigate additional fusion designs to further optimize recombinant TB vaccine production and performance.

Our results demonstrated that CPQ liposomes effectively present the His-tagged ESAT(Int7)CFP and EsxH(Int7)EsxG fusion proteins, leading to robust IgG antibody titers and strong antigen-specific IFN-γ secretion from splenocytes in immunized mice. These findings suggest that Int7-linked TB antigens adjuvanted with CPQ liposomes represent a promising next-generation platform for TB subunit vaccines.

However, in Mtb aerosol challenge experiments, the protective efficacy conferred by the CPQ vaccines remained partial when compared to BCG. This is consistent with outcomes observed from other protein subunit vaccines, which often fall short of the broad-spectrum protection elicited by live attenuated BCG. Given that BCG comprises a wide array of mycobacterial antigens, it induces diverse innate and adaptive immune responses that are difficult to replicate using a limited set of recombinant proteins. Nevertheless, the complexity of BCG makes it difficult to deconvolute which specific antigens drive its protective efficacy (82). Another limitation of this challenge study is the difficulty in comparing the consistency of immune responses across the groups owing to unequal sample sizes between the BCG and immunized groups. Although the CPQ/ESAT(Int7)CFP immunized groups exhibited a smaller standard deviation than the BCG group, this comparison is constrained by the differing numbers of biological replicates (n = 5 for BCG *versus* n = 4 for the fusion protein group). Future studies will address this limitation by using balanced group sizes and independent biological replicates to more rigorously assess response consistency across vaccine formulations. With respect to dose optimization, although we have demonstrated that antigen doses of 0.5, 1.5, and 2 μg can confer protective efficacy, future studies will explore higher antigen doses (>2 μg) to determine whether further improvements in protective immunity can be achieved.

Although ESAT(Int7)CFP and EsxH(Int7)EsxG were not directly compared in the present study, the primary objective was not to identify a single superior fusion antigen, but rather to develop a vaccine strategy that could lead to future incorporation of multiple protective TB antigens to maximize efficacy. In this context, the fusion proteins are intended to serve as complementary components within a broader vaccine formulation rather than as competing candidates. Future studies will therefore directly compare these fusion antigens and evaluate combined formulations to determine whether multivalent incorporation can further enhance protection against TB.

No overt adverse reactions or behavioral abnormalities were observed following intramuscular administration of CPQ-formulated vaccines in this study, and previous studies using CPQ-based TB (75) formulations similarly demonstrated favorable tolerability profiles in mice. Recently, additional CoPoP formulations for RSV (83) and herpes zoster vaccine (84) have proceeded into clinical testing, suggesting the safety of the vaccine platform. However, the present work did not include a formal assessment of local reactogenicity or tissue inflammation at the injection site nor systemic toxicity. Future studies incorporating histopathological analysis, quantitative inflammatory measurements, and longitudinal safety monitoring will therefore be important to more comprehensively define the local and systemic safety profile of CPQ-based tuberculosis vaccine formulations.

The CPQ liposomal platform offers flexibility in antigen inclusion, formulation, and immune tailoring. Future improvement of this system could enhance protective efficacy by (1) increasing antigen dose (2), incorporating additional TB antigens to broaden immune coverage, and (3) optimizing adjuvant combinations to amplify innate immune activation.

In summary, Int7-linked fusion antigens adjuvanted with CPQ liposomes offer a scalable, cost-effective, and immunogenic alternative to conventional TB vaccines, with the potential to be rapidly adapted for protection against emerging Mtb strains as well as other infectious diseases.

## Experimental procedure

### Design of the recombinant proteins

The pET-3a plasmids containing our engineered constructs were manufactured by GenScript. The BL21 (DE3) *E*. *coli* strain was obtained from Intact Genomics. The target proteins were designed as hybrid molecules consisting of primary sequences corresponding to ESAT6, CFP10, EsxG, and EsxH proteins of *M*. *tuberculosis* (strain ATCC 25618/H37Rv), retrieved from UniProtKB/Swiss-Prot database (accession numbers: P9WNK7, P9WNK5, O53692, and P9WNK3, respectively). The designs included a 6-histidine tag at either the C- or N-terminus of the single protein or the fusion protein. For Int7 fusion proteins, a 7-histidine linker is added between the proteins to mediate the connection between the two proteins (Table S1).

### Expression of the soluble recombinant proteins

An *E*. *coli* DirectPlate BL21 (DE3) chemically competent cells (Intact Genomics, Cat# 1019-36) clone harboring the target plasmid was streaked on a LB plate with 100 μg/ml Ampicillin (Teknova, Cat# L1093) and incubated at 37 °C for 16 h. A single colony was selected from the LB plate and grown in 2 ml of trypticase soy broth (BD Diagnostics, Cat# 292770) supplemented with 100 μg/ml Ampicillin (Quality Biological, Cat# 351-344-731) for 3 h. The resulting culture was inoculated into 50 ml of Terrific Broth (Gibco, Cat# A1374301) with 100 μg/ml Ampicillin and incubated at 37 °C until OD_600_ reached 1.0 to 1.2. Isopropyl-β-D-thiogalactopyranoside (IPTG) (Invitrogen, Cat# 15529019) was added to a final concentration of 1 mM for 3 h of induction at 37 °C. The cell pellet was collected after centrifugation at 4,000*g* for 30 min.

The pellet was lysed by incubation in B-PER Complete Bacterial Protein Extraction Reagent (Thermo Scientific, Cat# PI89822) according to the manufacturer’s instructions. The lysate was centrifuged at 16,000*g* for 20 min to remove cell debris, and the supernatant was collected for further protein purification steps. The lysate was mixed with 1X volume of Binding Buffer (50 mM Na_2_HPO_4,_ 300 mM NaCl, 10 mM Imidazole, pH 8.0). A total of 0.3 ml of Ni-NTA resin (Thermo Scientific, Cat# 88221) was packed into a 5 ml column, rinsed with 1 ml of DI H_2_O, and equilibrated with 1 ml of Binding Buffer. The lysate was then added to the column and passed through it ten times. After washing with 25 ml of Wash Buffer (50 mM Na_2_HPO_4,_ 300 mM NaCl, 20 mM Imidazole, pH 8.0), the target protein was eluted by adding 0.6 ml of Elution Buffer (50 mM Na_2_HPO_4,_ 250 mM NaCl, 300 mM Imidazole, pH 8.0) three times. The eluted fractions were collected and dialyzed against 1 L of PBS three times. Lastly, the purified proteins were filtered through a 0.22 μm membrane and examined using SDS-PAGE analysis. Protein concentrations were determined by using a BCA assay.

### Thermal stability assay

Nano-DSF thermal unfolding experiments were performed on a Prometheus NT.48 system (Nanotemper). Proteins were diluted to 5.625 μM in 1X PBS pH 7.4 and 10 μl of each protein was loaded into a capillary. The samples were heated from 20 °C to 95 °C at a rate of 1 °C/min. The intrinsic fluorescence signals at 330 nm and 350 nm were measured for each experiment. The T_m_ was calculated automatically based on the first derivative of fluorescence signal by the software PR.Thermcontrol (Nanotemper). The T_m_ values reported in this study were extrapolated from the first derivative of 330 nm fluorescence signal.

### Liposome preparation

Liposomes were prepared using ethanol injection and nitrogen-pressurized lipid extrusion as reported previously (64). The chemicals used for liposome synthesis are 1,2-dioleoyl-sn-glycero-3-phosphocholine (DOPC) (Corden, Cat# LP-R4-070), cholesterol (PhytoChol) (Wilshire Technologies, Cat# 57-88-5), Monophosphoryl Hexa-acyl Lipid A, 3-Deacyl (PHAD-3D6A) (Avanti Polar Lipids, Cat# 699855), QS-21 (Desert King), CoPoP and porphyrin-phospholipid (PoP) were produced as described previously (85, 86). The prepared liposomes were dialyzed against PBS twice at 4 °C to remove ethanol and passed through a 0.22 μm sterile filter. The final liposome concentration was adjusted to 320 μg/ml and stored at 4 °C. Liposome sizes and polydispersity index were determined by dynamic light scattering (DLS) with a NanoBrook 90 plus PALS instrument after 500-fold dilution in PBS. The CoPoP/PHAD (CP) liposomes had a mass ratio of [DOPC:CHOL:CoPoP:3D6A-PHAD] [20:5:1:0.4], PoP/PHAD (2HP) liposomes, which have a similar formulation as CP but contain hydrogen instead of cobalt in the PoP, served as the control liposomes, having a mass ratio of [DOPC:CHOL:PoP:3D6A-PHAD] [20:5:1:0.4]. For liposomes containing QS-21, QS-21 (1 mg/ml) was added to the liposomes after formation at an equal mass ratio as PHAD. The CoPoP/PHAD/QS-21 (CPQ) had a mass ratio of [DOPC:CHOL:CoPoP:3D6A-PHAD:QS-21] [20:5:1:0.4:0.4], PoP/3D6A-PHAD/QS-21 served as the control liposomes which had a mass ratio of [DOPC:CHOL:PoP:3D6A-PHAD:QS-21] [20:5:1:0.4:0.4].

### Ni-NTA bead competition assay

To assess recombinant protein binding stability in particle form, Ni-NTA magnetic beads (ThermoFisher, Cat# 88831) were added to liposome-incubated antigens or free proteins in PBS. Proteins (80 μg/ml) were mixed with an equal volume of CPQ liposomes (CoPoP concentration: 320 μg/ml) and incubated at room temperature for 3 h to allow particle formation. Subsequently, the samples were incubated with Ni-NTA magnetic beads for 30 min at room temperature. Following incubation, the supernatant and magnetic beads were separated and collected using a magnetic separator (ThermoFisher, Cat# 12321D). The beads were then resuspended in PBS. Denaturing reducing loading dye (Thermo Scientific, Cat# 39000) was then added to all samples (supernatant and beads) and heated at 95 °C for 10 min. A total of 46 μl samples (1.5 μg protein) were loaded into each well of a Tris-Glycine gel (Bio-Rad, Cat# 4568084). PageRuler Prestained Protein Ladder (4 μl) (Thermo Scientific, Cat# 26616) was also loaded. Gels were run at a constant 200 V for 30 min and stained with SimplyBlue SafeStain (Invitrogen, Cat# LC6065) following the manufacturer’s instructions. Gel images were acquired using a Bio-Rad GelDoc Go Imaging System.

### Slot blot assay

A 0.2 μm nitrocellulose membrane (Thermo Scientific, Cat# 88013) was fixed in a 48-well microfiltration apparatus (Bio-Rad, Cat# 170-6545). Initially, 50 μl of PBS was loaded into each well, followed by 50 μl of each sample (50 ng), which flowed through the membrane by gravity. After blocking with 5% bovine serum albumin (VWR, Cat# 97061-422) at room temperature for 1 h, the membrane was incubated with the anti-ESAT6 serum (BEI Resources, NR-13803), anti-CFP10 serum (BEI Resources, NR-13801), or anti-His-tag monoclonal antibody (GenScript, Cat3 A00186) at a 1:1000 dilution in 5% BSA for 1 h at 37 °C. After three additional washes with PBS, the membrane was incubated with anti-rabbit IgG antibody HRP (GenScript, Cat# A01827) or anti-mouse IgG antibody HRP (Cell Signaling, Cat# 7076S) at a 1:1000 dilution in 5% BSA for 1 h at 37 °C. After three additional washes with PBS, VisiGlo HRP substrate mixture (VWR, Cat# 97063-148) was added to the membrane, and images were captured by Bio-Rad ChemiDoc Imager.

### Fluorescence quenching assay

The recombinant protein (200 μg) was dialyzed into 100 mM sodium bicarbonate buffer (pH 9.0) using a Slide-A-Lyzer Dialysis Cassette (Thermo Scientific, Cat# 66383) twice at 4 °C. The protein was then labeled with 10 μl of 1 mg/ml DY-490-NHS-Ester (Dyomics, Cat# 490-01) for 1 h at room temperature with constant mixing. The solution was subsequently dialyzed with PBS three times to remove free dye. To measure the fluorescent protein binding efficiency to the liposomes, the fluorescence of the labeled protein was detected with 491 nm excitation and 527 nm emission in a TECAN Safire microplate reader. Upon binding to the liposomes, the fluorescence is quenched due to energy transfer from DY-490-ESAT-CFP to the porphyrin of CPQ or 2HPQ. Binding kinetics were determined by monitoring the changes in fluorescence after incubating the same volume of protein (80 μg/ml) with liposomes in PBS (320 μg/ml) or PBS alone. Samples were diluted 10-fold prior to measurement in the plate reader. The binding percentage was calculated using the following equation:

$$\text{Binding}\%=1-\left(\frac{{\text{Fluorescence}}_{\text{test}\;\text{liposomes}}}{{\text{Fluorescence}}_{\text{free}\;\text{labeled}\;\text{protein}}}\right)\times 100\%$$

### Murine immunization study

Murine studies were performed according to protocols approved by the University at Buffalo IACUC. Six-to eight-week-old female CB6F1/J mice (The Jackson Laboratory, JAX stock #100007) received intramuscular injections on days 0 and 14 containing 2 μg ESAT-CFP or EsxH-EsxG proteins combined with CPQ liposomes. The commercial adjuvant Alum (InvivoGen, Cat# vac-alu-50) was used as a comparison. CPQ vaccines were prepared by incubating proteins (80 μg/ml) with liposomes (320 μg/ml) for 3 h at room temperature. Alum vaccines were prepared by mixing antigen with Alum diluted to 1 mg/ml in PBS. On day 28 post-immunization, blood was collected from mice under 5% isoflurane anesthesia, followed by humane euthanasia *via* cervical dislocation. Sera were separated by centrifuging the whole blood at 2000*g* for 20 min at 4 °C. Spleens were excised and passed through a cell strainer (VWR, Cat# 76327-100) using ice-cold PBS to isolate splenocytes.

### Antigen-specific antibody titer measurement

Anti-ESAT6, anti-CFP10, anti-EsxH, and anti-EsxG IgG titers were assessed by ELISA using 96-well plates coated with 1 μg/ml of ESAT6, CFP10, EsxH, or EsxG in coating buffer (3.03*g* Na2CO3; 6*g* NaHCO3 in 1 L deionized water, pH 9.6) for 1 h at 37 °C. After washing and blocking with 2% BSA in PBS containing 0.1% Tween-20 (PBS-T), serial dilutions of mouse sera in PBS were added and incubated for 1 h at 37 °C. After washing with PBS-T, HRP-linked anti-mouse IgG antibody (Cell Signaling, Cat# 7076S) was added, followed by 3,3′,5,5′-tetramethylbenzidine (TMB) substrate (Surmodics, Cat# TMBW-1000-01). The reaction was stopped with 1M HCl, and absorbance was measured at 450 nm. Titers were defined as the reciprocal serum dilution at which the absorbance exceeded the background by 0.5 absorbance units.

### ELISpot

A total of 3 × 10^5^ splenocytes were re-stimulated with 1 μg/ml of PepMix *M*. *tuberculosis* ESAT-6 (JPT, Cat# PM-MYCTU-ESAT6), PepMix *M*. *tuberculosis* CFP-10 (JPT, Cat# PM-MYCTU-CFP10), or PepMix *M*. *tuberculosis* TB10.4 (JPT, Cat# PM-MYCU-TB104) for 18 h at 37 °C in a mouse IFN-γ/IL-2 double-color ELISPOT plate (C.T.L., Cat# mu-IFN-γ/IL-2) according to the manufacturer’s instructions. Media alone was used as a negative control.

### *M*. *tuberculosis* challenge study

Six-to eight-week-old female CB6F1/J mice were purchased from the Jackson laboratory as described above and housed in a BSL2 at CSU during vaccinations. Studies were carried out using protocols approved by the Colorado State University IACUC. For BCG vaccination, mice were injected SubQ with 50 μl of 1 × 10^6^ CFU BCG Pasteur in the scruff of the neck. All other vaccinations were performed intramuscularly in the thigh region upon injection of 50 μl saline or 2 μg of CPQ/ESAT-CFP or 2 μg of CPQ/EsxH-EsxG. Three weeks later, mice were boosted with the corresponding priming vaccine, except BCG-vaccinated mice which received a saline boost. Mice were moved 3 weeks later into BSL3 conditions and infected *via* the aerosol route with a Glas-Col apparatus calibrated to deliver 50 to 100 CFUs/mouse of actively growing *M*. *tuberculosis* H37Rv ATCC 27294.

On day 0 and 30 post-infection, mice were humanely euthanized *via* CO_2_ asphyxiation and organs harvested to enumerate bacterial burden in lungs as well as for histopathological analysis. Lungs were homogenized at 7200 rpm for 16 s in PBS using 7 ml Precellys tubes and plated on 7H11 nutrient agar. After incubating for 3 to 4 weeks at 37 °C, CFUs were enumerated, log10 transformed and analyzed with Prism using one-way ANOVA and Tukey’s post-test in comparison to saline-vaccinated mice.

## Data availability

All data are included in the manuscript and supporting information.

## Supporting information

This article contains supporting information.

## Conflict of interest

The authors declare the following financial interests/personal relationships which may be considered as potential competing interests: Wei-Chiao Huang and Jonathan Lovell hold interest in POP Biotechnologies. Other authors have no conflicts.
