# Supplementary material for: Engineering internally his-tagged ESX secretion system fusion antigens for tuberculosis nanoparticle vaccines
Source: J Biol Chem. 2026 Jul 16;302(9):113337. doi: 10.1016/j.jbc.2026.113337 (PMC13476637; doi:10.1016/j.jbc.2026.113337)
Supplement: Supplementary file 1 [file mmc1.pdf]

Supporting Information for

Engineering internally His-tagged ESX secretion system fusion antigens for tuberculosis nanoparticle vaccines

Wen-Ling Hsu[1], Yang Jiao[1], Wei-Chiao Huang[1], Kristina N. Tran[3], Brennen T. Troyer[3], Matthew Hvasta[2], Brian Kuhlman[2], Andres Obregon-Henao[3*], Jonathan F. Lovell[1*]

[1]Department of Biomedical Engineering, University at Buffalo, State University of New York, Buffalo, USA. [2]Department of Biochemistry and Biophysics, University of North Carolina School of Medicine, Chapel Hill, North Carolina, USA. [3]Department of Microbiology, Immunology and Pathology, Colorado State University, Fort Collins, USA.

Corresponding Authors:

*Andres Obregon-Henao: Department of Microbiology, Immunology and Pathology, Colorado State University, Fort Collins, Colorado, USA; Email: Andres.Obregon@colostate.edu

*Jonathan F. Lovell: Department of Biomedical Engineering, University at Buffalo, State University of New York, Buffalo, New York 14260, USA; Email: jflovell@buffalo.edu

This PDF file includes:

Supplementary Figure 1
Supplementary Figure 2
Supplementary Figure 3
Supplementary Table 1

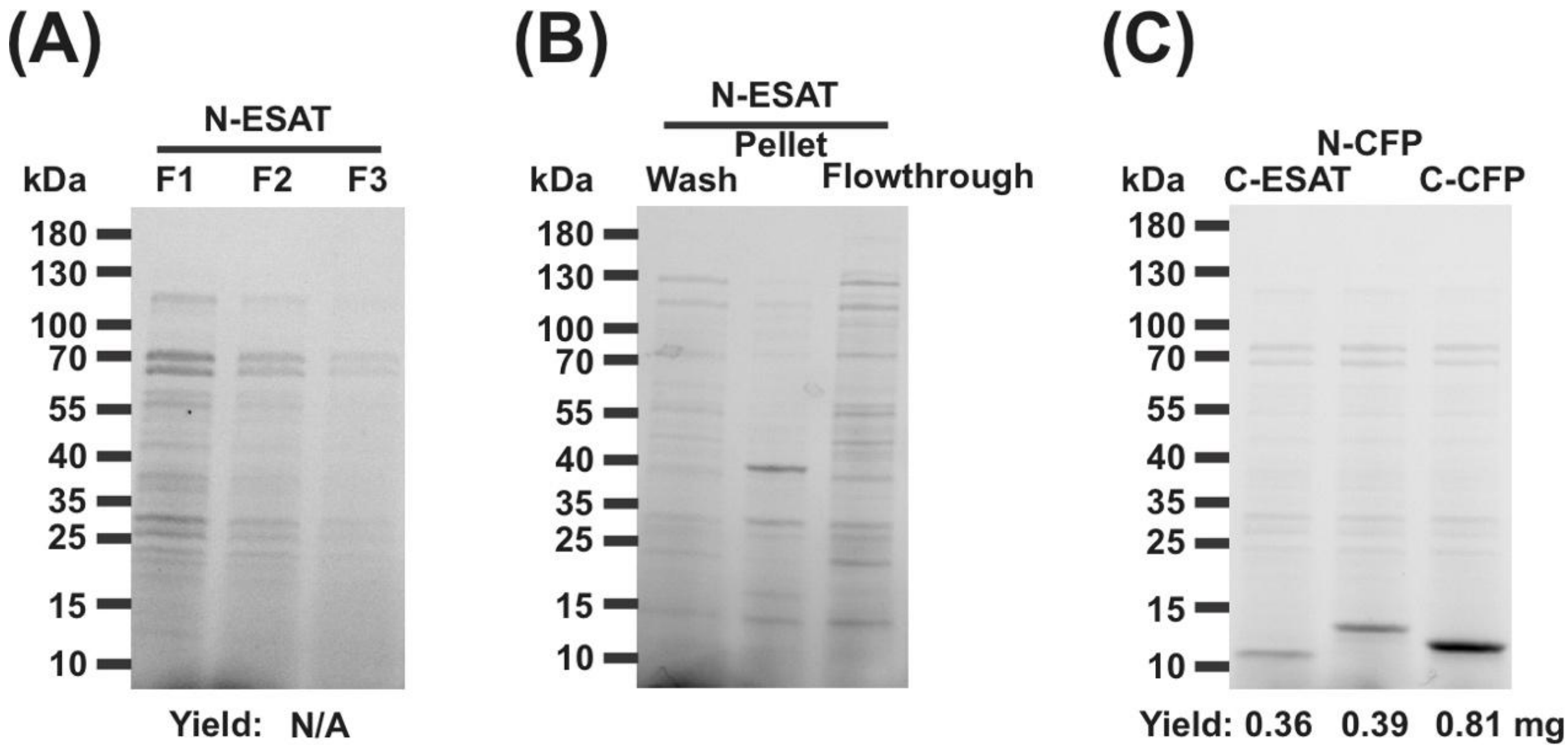

Supplementary Fig. 1: Soluble expression of recombinant ESAT6 and CFP10 proteins. (A) SDS-PAGE analysis of soluble fractions from recombinant ESAT6 containing a N-terminal hexahistidine tag (N-ESAT). The predicted molecular weight of N-ESAT is 11 kDa. No distinct N-ESAT band was detected following staining with SimplyBlue SafeStain (Invitrogen, Cat# LC6065). F1-F3, elution fractions 1-3. (B) SDS-PAGE analysis of wash, insoluble pellet, and flowthrough fractions from N-ESAT purification. N-ESAT was not detected in the wash fraction, insoluble pellet, or soluble lysate flowthrough. Wash, wash fraction; Pellet, insoluble pellet obtained after centrifugation of the cell lysate; Flowthrough, soluble supernatant collected after centrifugation of the cell lysate. (C) Soluble expression of ESAT6 containing a C-terminal hexahistidine tag (C-ESAT) and CFP10 containing either N- or C-terminal hexahistidine tags (N-CFP and C-CFP, respectively). The predicted molecular weights of C-ESAT, N-CFP, and C-CFP are 11, 12, and 12 kDa, respectively. Protein yields were quantified by BCA assay following purification.

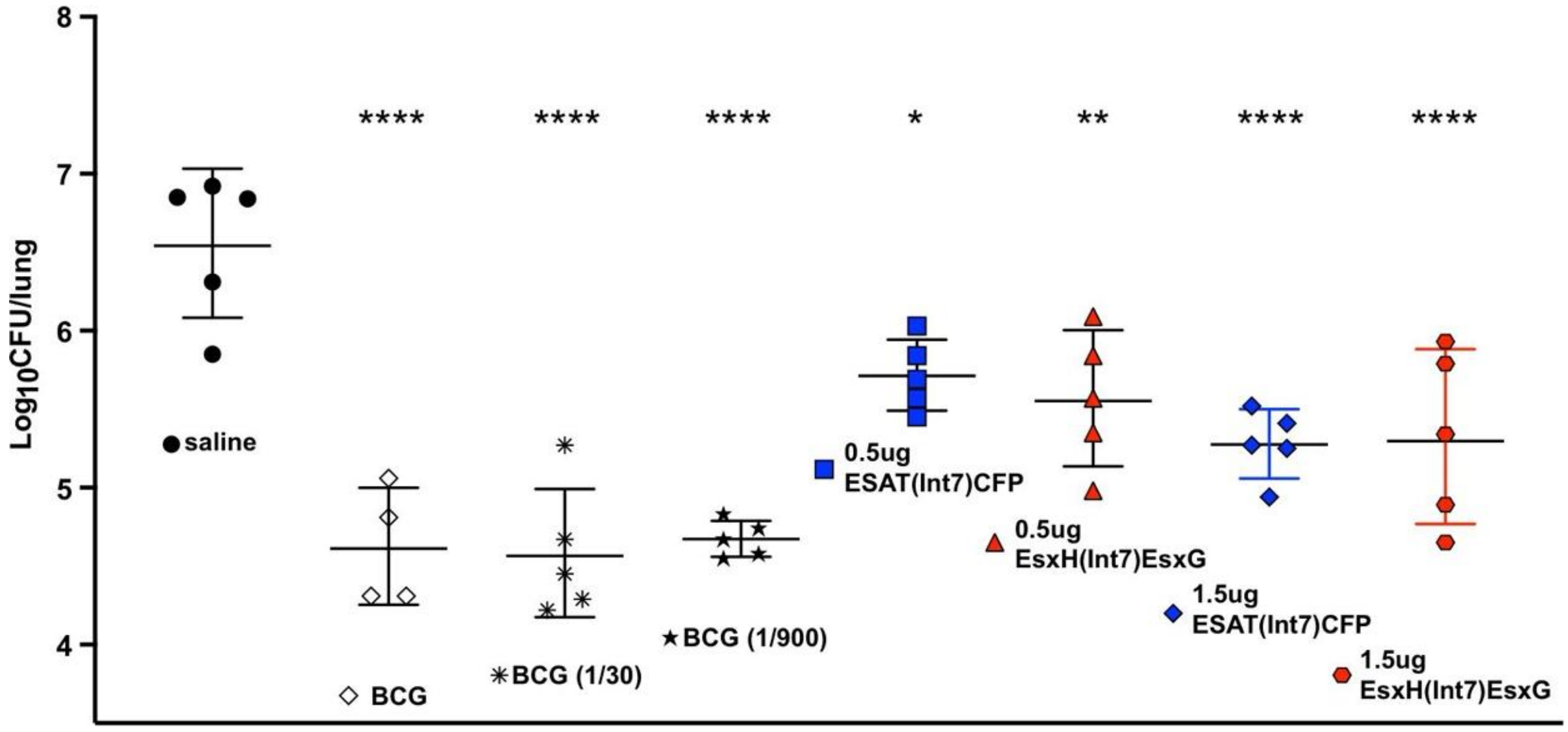

Supplementary Fig. 2: Dose optimization of BCG, CPQ/ESAT(Int7)CFP, and CPQ/EsxH(Int7)EsxG in *M. tuberculosis* challenge murine model. Lung colony-forming units (CFUs) were quantified following immunization with BCG (administered at the dose described in Methods or diluted 30- or 900-fold in saline), CPQ/ESAT(Int7)CFP (0.5 or 1.5 μg antigen), or CPQ/EsxH(Int7)EsxG (0.5 or 1.5 μg antigen). Graphs show mean ± std. dev. for n=5 mice per group (n=4 for the BCG group). Statistical significance was determined by one-way ANOVA followed by Dunnett's multiple-comparison test. *$p < 0.05$, **$p < 0.01$, ***$p < 0.005$, and ****$p < 0.001$.

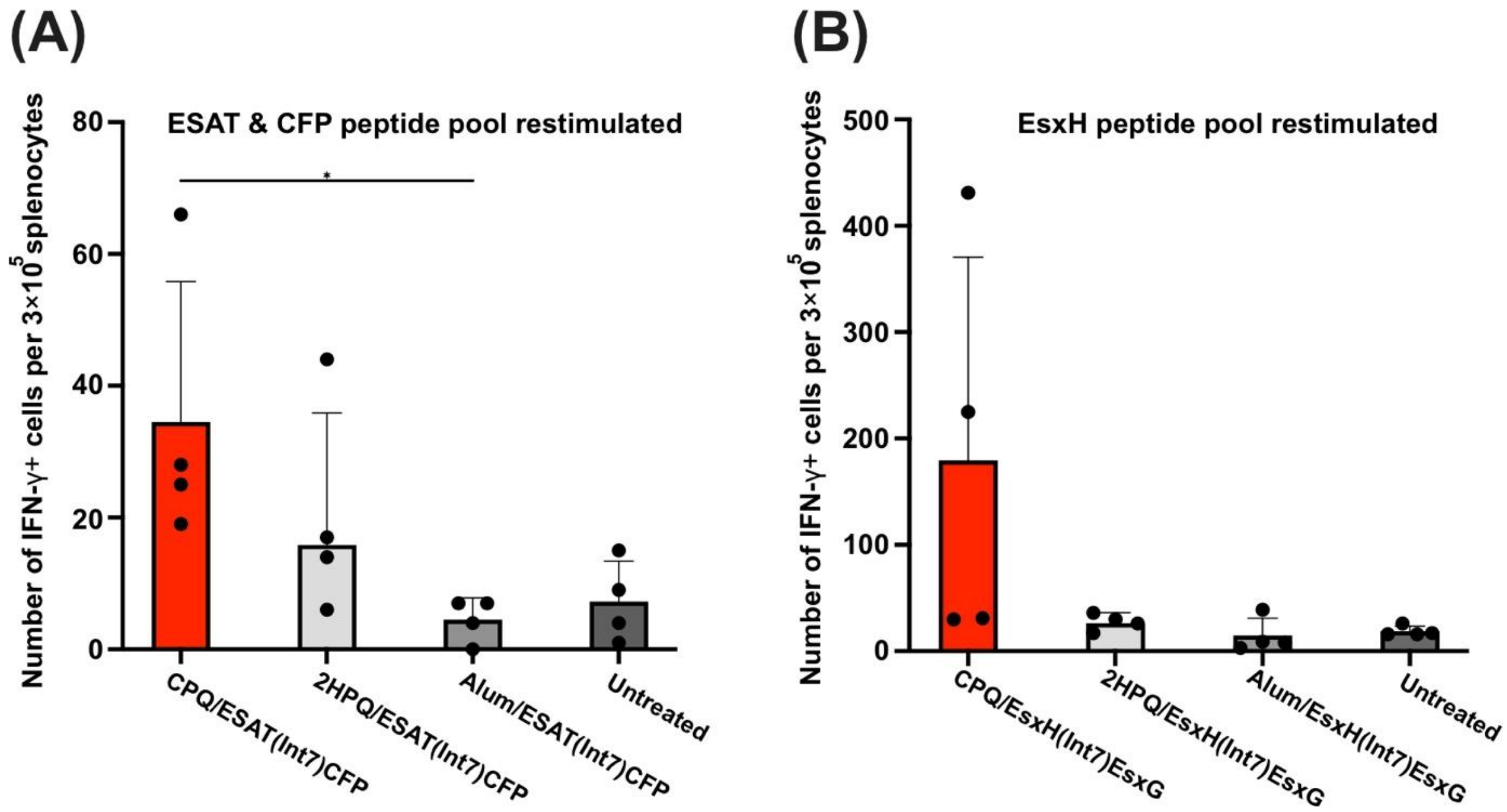

Supplementary Fig. 3: CPQ vaccines induce stronger antigen-specific T cell responses than Alum- or 2HPQ-adjuvanted vaccines in mice. CB6F1/J mice (n = 4 per group) were immunized intramuscularly with ESAT(Int7)CFP or EsxH(Int7)EsxG vaccines (2 µg antigen) on days 0 and 14. Splenocytes were harvested 14 days after the second immunization and restimulated with ESAT6 and CFP10 peptide pools mixed at a 1:1 concentration ratio or with EsxH peptide pool. (A) ESAT6- and CFP10-specific T cell responses were quantified by mouse IFNγ single-color ELISPOT assay (C.T.L., Cat# mu-IFN-γ). Experimental groups included untreated, Alum/ESAT(Int7)CFP, 2HPQ/ESAT(Int7)CFP, and CPQ/ESAT(Int7)CFP (n = 4 per group). (B) EsxH-specific T cell responses were quantified by mouse IFNγ single-color ELISPOT assay (C.T.L., Cat# mu-IFN-γ). Experimental groups included untreated, Alum/EsxH(Int7)EsxG, 2HPQ/EsxH(Int7)EsxG, and CPQ/EsxH(Int7)EsxG (n = 4 per group). Data are presented as geometric mean ± geometric SD. Statistical significance was determined by one-way ANOVA followed by Tukey’s multiple-comparison test. Significance levels are indicated as *$p < 0.05$, **$p < 0.01$, ***$p < 0.005$, and ****$p < 0.001$.

Supplementary Table 1: Sequences used in this study

Construct	Sequence
C-CFP	MAEMKTDAATLAQEAGNFERISGDLKTQIDQVESTAGSLQGQWRGAAGTAAQAAVVRFQEAANKQKQELDEI<br>STNIRQAGVQYSRADEEQQQALSSQMGFHHHHHH
C-ESAT	MTEQQWNFAGIEAAASAIQGNVTSIHSLLDEGKQSLTKLAAAWGGSGSEAYQGVQQKWDATATELNNALQNL<br>ARTISEAGQAMASTEGNVTGMFAHHHHHH
C-ESAT-CFP	MTEQQWNFAGIEAAASAIQGNVTSIHSLLDEGKQSLTKLAAAWGGSGSEAYQGVQQKWDATATELNNALQNL<br>ARTISEAGQAMASTEGNVTGMFAGGGGSGGGGSGGGGSMAEMKTDAATLAQEAGNFERISGDLKTQIDQVES<br>TAGSLQGQWRGAAGTAAQAAVVRFQEAANKQKQELDEISTNIRQAGVQYSRADEEQQQALSSQMGFHHHHHH
ESAT(Int7)CFP	MTEQQWNFAGIEAAASAIQGNVTSIHSLLDEGKQSLTKLAAAWGGSGSEAYQGVQQKWDATATELNNALQNL<br>ARTISEAGQAMASTEGNVTGMFAHHHHHHHMAEMKTDAATLAQEAGNFERISGDLKTQIDQVESTAGSLQGQ<br>WRGAAGTAAQAAVVRFQEAANKQKQELDEISTNIRQAGVQYSRADEEQQQALSSQMGF
N-ESAT-CFP	HHHHHHMTEQQWNFAGIEAAASAIQGNVTSIHSLLDEGKQSLTKLAAAWGGSGSEAYQGVQQKWDATATELN<br>NALQNLARTISEAGQAMASTEGNVTGMFAGGGGSGGGGSGGGGSMAEMKTDAATLAQEAGNFERISGDLKTQ<br>IDQVESTAGSLQGQWRGAAGTAAQAAVVRFQEAANKQKQELDEISTNIRQAGVQYSRADEEQQQALSSQMGF
C-EsxH	MSQIMYNYPAMLGHAGDMAGYAGTLQSLGAEIAVEQAALQSAWQGDTGITYQAWQAQWNQAMEDLVRAYHAM<br>SSTHEANTMAMMARDTAEAAKWGGHHHHHHH
C-EsxG	MSLLDAHIPQLVASQSAFAAKAGLMRHTIGQAEQAAMSAQAFHQGESSAAFQAAHARFVAAAAKVNTLLDVA<br>QANLGEAAGTYVAADAAAASTYTGFHHHHHHH
EsxH(Int7)EsxG	MSQIMYNYPAMLGHAGDMAGYAGTLQSLGAEIAVEQAALQSAWQGDTGITYQAWQAQWNQAMEDLVRAYHAM<br>SSTHEANTMAMMARDTAEAAKWGGHHHHHHHMSLLDAHIPQLVASQSAFAAKAGLMRHTIGQAEQAAMSAQA<br>FHQGESSAAFQAAHARFVAAAAKVNTLLDVAQANLGEAAGTYVAADAAAASTYTGF
EsxG(Int7)EsxH	MSLLDAHIPQLVASQSAFAAKAGLMRHTIGQAEQAAMSAQAFHQGESSAAFQAAHARFVAAAAKVNTLLDVA<br>QANLGEAAGTYVAADAAAASTYTGFHHHHHHHMSQIMYNYPAMLGHAGDMAGYAGTLQSLGAEIAVEQAALQ<br>SAWQGDTGITYQAWQAQWNQAMEDLVRAYHAMSSTHEANTMAMMARDTAEAAKWGG
